# COVID-19 in Africa: rethinking the tools to manage future pandemics

**DOI:** 10.4314/ahs.v21i4.3

**Published:** 2021-12

**Authors:** Ismaila Emahi, Mimmie CNC Watts, Samuel Azibere, Joseph F Morrison, Kwabena AN Sarpong

**Affiliations:** 1 Department of Chemical Sciences, University of Energy and Natural Resources, Sunyani, Ghana; 2 Regional Centre for Energy and Environmental Sustainability (RCEES), Sunyani, Ghana; 3 College of Health, Federation University, Melbourne Australia; 4 Tris Pharma, Inc, NJ, USA; 5 Department of Biochemistry, Cell and Molecular Biology, University of Ghana, Legon, Ghana; 6 West African Centre for Cell Biology of Infectious Pathogens, University of Ghana, Legon, Ghana

**Keywords:** Covid-19, Ebola, Science Leadership in Africa, Vaccine, SARS-COV-2, Preventative Health, Future Pandemics, Health leadership

## Abstract

Corona virus disease 2019 (COVID-19) remains an incurable, progressive pneumonia-like illness characterized by fever, dry cough, fatigue, and headache during its early stages. COVID-19 has ultimately resulted in mortality in at least 2 million people worldwide. Millions of people globally have already been affected by this disease, and the numbers are expected to increase, perhaps until an effective cure or vaccine is identified.

Although Africa was initially purported by the World Health Organization (WHO) to be severely hit by the pandemic, Africa recorded the least number of cases during the first wave, with lowest rates of infections, compared to Asia, Europe, and the Americas. This statistic might be attributed to the low testing capacity, existing public health awareness and lessons learnt during Ebola epidemic. Nonetheless, the relatively low rate of infection should be an opportunity for Africa to be better prepared to overcome this and future epidemics.

In this paper, the authors provide insights into the dynamics and transmission of the severe acute respiratory syndrome corona virus (SARS-CoV-2) during the first wave of the pandemic; possible explanations into the relatively low rates of infection recorded in Africa; with recommendations for Africa to continue to fight Covid-19; and position itself to effectively manage future pandemics.

## Introduction

Africa fared well during the first wave of the Covid19 Pandemic, since it was declared a global Pandemic by the World Health Organization (WHO) on March 12, 2020 with Wuhan, China the epicentre of the virus disproportionately affected^1^. At the time of the declaration, the spread of the Covid19 vius was mainly confined in Asia (mainly China) a few countries in Europe (UK, France, Germany) and North America (USA) with travellers the most affected. However, the last twelve months has seen a shift in the spread of the Covid19 virus globally, with the UK, Italy, the United States of America, and India overly affected. Unlike other parts of the world, Africa seems to be faring well, despite initial concerns by other countries on Africa's ability to manage the Covid19 pandemic at outset, while still recognising the impact of the second wave on Africa, comparatively. Regardless, Covid19 is not the first, nor the most recent pandemic in living memory including the Severe Acute Respiratory Syndrome (SARS) and Ebola.

In November 2002, an outbreak of severe acute respiratory syndrome (SARS) was reported to have started in Foshan in the Guangdong province of China 2. This epidemic lasted for approximately eight months and affected over 8,000 people worldwide, killing nearly 800^3^. Africa was essentially spared of this epidemic albeit South Africa recording one case, which resulted in the death of the affected individual^3^. Another SARS outbreak originated in Wuhan (Hubei Province, China) in December 2019 and caused significant morbidity and mortality worldwide. Both SARS outbreaks were caused by corona viruses and have been designated SARS-CoV-1 and SARS-CoV-2 respectively for the 2002 and 2019 outbreaks. However, the World Health Organization (WHO) learned that the 2019 SARS-causing corona virus was a completely new strain, giving rise to the name 2019-nCoV or Novel Coronavirus 2019. Nonetheless, SARS-CoV-2 remains the name of choice by the International Committee on Taxonomy of Viruses 4 and the current name used by nearly all researchers and the media alike. The disease caused by SARS-CoV-2 was named by the WHO and continues to be called COVID-19, basically implying Coronavirus disease of 2019. A lot remains unknown about the “spread” and dynamics of the virus although significant knowledge has been acquired since the pandemic first broke out. Infection with the virus (SARS-CoV-2), can be asymptomatic or can result in mild to severe symptomatic disease (COVID- 19) 5.

As at January 31, 2021, there has been approximately 103.5 million confirmed COVID-19 cases worldwide, and about 2.2 million deaths (Table 1). Africa has not been spared this time. So far over 3.5 million confirmed COVID-19 cases and more than 91,000 resulting deaths have been recorded across the entire African continent, with South Africa being the worst hit with over 1.4 million cases and 44,164 cumulative deaths as at January 31, 2021. Nonetheless, the African continent remains relatively low-hit by this pandemic (Table 1), which raises the question about how Africa, with very limited and subpar healthcare systems, compared to the advanced healthcare facilities in industrialized countries has not been badly hit as initially anticipated, even when we reflect on the impact of the second wave.

**Table 1:** Comparison of the Covid-19 situation in Africa compared to other regions

Covid-19 Statistics	Global	Asia	North America	Europe	Africa
Population	7,795,232,630	4,641,054,775 59.54%	592,072,212 7.60%	747,636,026 9.56%	1,340,598,147 17.20%
Cases	103,117,645	23,020,850 22.32%	30,485,901 29.56%	30,152,779 29.24%	3,569,944 3.46%
Recovered	74,758,047	21,458,441 28.7%	19,349,287 25.88%	16,865,386 22.56%	3,045,741 4.07%
Deaths	2,227,912	371,732 16.69%	650,182 29.18%	699,751 31.41%	90,554 4.06%
Case Fatality Rate (%)	2.16	1.61	2.13	2.32	2.54

Reference: www.worldometers.info/coronavirus – accessed February 1, 2021

There are considerable, continuing efforts throughout the world to develop effective, but safe drugs, and precise vaccines for SARS-CoV-2. Several countries have already begun vaccinating individuals with mRNA-based vaccines in addition to over 100 vaccines at preclinical stages and clinical stages^6^. Indeed, inoculations commenced in the UK and USA in January 2021, with Australia, one of the least hit Covid-19 nations set to follow suit in March 2021. Meanwhile, several countries across the globe, particularly in the United States are experiencing a second wave of the pandemic with associated huge numbers of fatalities. This is enough indication that the pandemic is not yet over, and Africa cannot yet claim a successful defeat to the virus. In fact the second wave of this pandemic could be devastating for Africa, if poorly managed. African leaders must therefore be better prepared. The recent availability of vaccines for Covid19 has highly another issue for Africa and developing countries, inequities, and access. Presently, the challenging factor for Africa remains the denial of accessibility of the vaccines to many countries in Africa, as it is believed that most of the vaccines are reserved for wealthy countries^39^. While not within the scope of this paper, vaccine inequity is an issue, we aim to write a separate paper on. This paper seeks to explore some possible explanations, tools and make recommendations for Africa, how to continue managing the second wave of the Covid-19 pandemic, and effectively controlling similar infectious diseases pandemics in the future.

## Dynamics and Transmission of Corona viruses

Six types of coronaviruses have been identified as human pathogens. HCoV-NL63 and HCoV-229E are Alphacoronavirus (alphCoV) types whereas HCoV-OC43, HCoVHKU1, SARS-CoV (including SARS-CoV 1 and 2), and MERS-CoV belong to the Betacoronavirus (betaCoV) type 7. SARS-COV-2 is a single-stranded RNA with 26 – 32 kb genome size containing 6 – 11 open reading frames (ORFs). The ORFs encode 16 non-structural proteins (nsps), structural, and accessory proteins 8. Phylogenic analyses suggest that SARS-CoV-2 is closely related to Bat coronavirus and Bat SARS-like CoV than SARS-CoV and MERS-CoV^9^, ^10^. Although the SARS-CoV-1 and MERS coronaviruses cause severe illnesses similar to the SARS-CoV-2, the case fatality rates of SARS-CoV-2 is estimated to be relatively lower 11 compared to SARS-CoV-1 (9%) and MERS-CoV (36%) 12. The transmission of both SARS-CoV and MERS-CoV, however, remained very limited^13^, ^14^ unlike the SARS-CoV-2, which has spread to nearly every corner of the globe and has lasted, so far, for a little over a year. The transmission of SARS-COV-2 has been likened to that of pandemic influenza due to its rapid surge across the globe and likelihood for it to circulate seasonally 14, 15. Liu et al (2020)^16^ have indicated that patients with severe Covid-19 usually have high viral loads and long virus-shedding period, which was also reminiscent of the 2002–03 SARS-CoV-1 infections. The viral load has been shown to peak during the first 5–7 days of infection and gradually drops afterwards^17^. This may therefore account for the reasons why some Covid-19 patients exhibit severe symptoms while others remain asymptomatic and may even recover from the illness without any indication of having acquired the virus.

Whereas a patient with a high viral load infection could end up in intensive care and possible death from the virus, individuals with low viral load may remain ambulant, exhibit no signs or symptoms of the disease until their bodies eventually clear the virus. We term this later case, automatic recovery. Since no health issues were experienced, no medical assistance is sought throughout the infection and incubations. This automatic recovery hypothesis could possibly account for the overall low detection rates of Covid-19 infections in Africa, which could be due to several underlying reasons, discussed in section 3.0. Nonetheless, such asymptomatic carriers, if unidentified and treated on time, could unexpectedly increase the rate of spread of the virus^18^.

## Insight into the relatively low Covid-19 cases during the first wave in Africa

The African continent appears to have managed Covid-19 better or at least recorded the least number of cases from the Covid-19 disease despite its comparatively subpar health care systems (Table 1).

**Figure 1:**
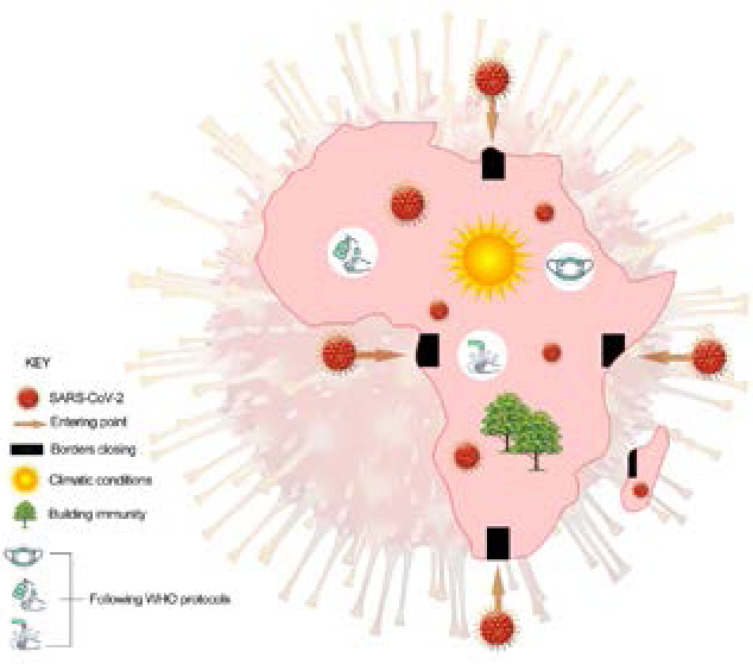
Summary of key interventions adopted by Africa to manage the Covid-19 pandemic. First case of the virus, recorded in Egypt, was imported. Gradually, the virus found its way to several other countries. The subsequent closing down of borders ensured that the spread of the virus was curtailed. Other factors that may have contributed to Africa's lower number of cases include climatic conditions (largely, very warm weather) that may not be favourable for the virus to thrive; strictly enforcing the recommended WHO preventative guidelines, as well as availability of herbal medicines and diets used by Africans to boost their immune systems.

For a continent that accounts for approximately 17 percent of the world's population, Africa has so far contributed only about 3.5 % in the total reported cases and 4 % in deaths resulting directly from Covid-19. It is arguable that the data reported from Africa may be inaccurate or underreported, but similar questions could be asked about the global data reported as there are several countries outside of Africa that are likely underreporting or deliberately misreporting. Issues of data rporting and low testing capacities therefore introduce some complexity into the argument, which is beyond the scope of this article. So, did Africa really manage the pandemic better or it probably survived it as a matter of sheer luck? Sections 3.1 through to 3.7 provides insights into this curiosity, also summarized in Figure 1.

## Climatic factors

Climatic factors have largely been attributed to the low prevalence and incidence of the Covid-19 virus in Africa. The negative effect of high temperatures and increased humidity on the spread of Covid-19 has been well documented 5, 19–25. Generally, while the corona viruses cannot survive for long hours on warm surfaces, cold temperatures and low humidity provide very conducive environments for the survival of the virus. In fact, the origin and time of the outbreak of both SARS-COV-1 and SARS-COV-2 have been further explained to account for the environmental effect on the transmission of the virus 26. SARS-COV-1, which broke out from Foshan, Guangdong Province occurred in November. The SARS-COV-2 broke out in December in Wuhan, Hebei Province. November and December are reportedly the coldest months in China with these two cities (Foshan and Wuhan) recording the lowest humidity conditions in the respective years the corona virus broke out 26. This suggests that low humidity and low temperatures at the time of both outbreaks, provided conducive conditions for the virus. The argument is however, debatable as similar viral breakouts could have been expected in China every year during winter times. Despite a few other works that did not find any correlation between high temperature and humidity with transmission of SARS-COV-2 27, 28, the global picture suggests that the transmission of the virus is significantly slowed in high temperature and high humidity environments. This therefore implies that the environmental conditions of several countries in Africa are protective for the widespread /transmission of the virus.

Certainly, these environmental factors alone cannot account for most of the variability in the transmission of Covid-19, just like the considerable variability in climate conditions throughout Africa. A climate-dependent modelling of the Covid-19 pandemic suggests that a key factor in the transmission of the virus is lack of population immunity rather than climate 29. Whereas climate may play a complex role, the authors note that a summer or warm weather alone, may not necessarily reduce the rate of spread or transmission of Covid-19^29^.

## Youthful population and low population density

Africa has a young population with the average age on the continent being 19.7 years compared to Europe's 43 years^30^. Age is undoubtedly a strong indicator of disease onset, and severity of Covid-19, with the mortality rate of Covid-19 in younger people, significantly lower 31. Perhaps a better explanation to the lower rate of spread and decreased severity of Covid-19 in Africa, is Africa's youthful population compared to the rest of the world. With a median age of 19.7 years most people who contract Covid-19 may be young enough and able to withstand some of the opportunistic infections from the virus. This may have translated into the asymptomatic cases and decreased severity of the disease in Africa.

The low population density coupled with the fact that older folks in Africa tend to live on their own in rural areas may be another factor. This is unlike developed countries where senior citizens may live-in nursing homes, and retirement villages or other residential facilities. Nursing home residents have experienced the worse outcomes in terms of incidence and prevalence of Covid-19 overall 32. This could be due to closer proximity and more physical contact between residents. Employees working across multiple aged care facilities, and private residents act as vectors in the spread of the virus, as seen in Victoria, Australia, during the second wave^33^. Further, the elderly living in residential facilities may also suffer from other co-morbidities, a common scenario in developed countries where modern medicine has contributed to prolonged life, sometimes at the expense of the quality.

Another benefit of the youthful population of Africa is that young people generally spend more time outdoors, in the sun- the benefit of this sun exposure being an increase in the production of vitamin D, an essential micronutrient that could reduce Covid-19 infections and related deaths 34, 35.

## Border Closures, Lockdowns and Restricted Public Gathering

Quick and stringent border controls, lockdowns, reduction in community gatherings, with mandatory use of facemask outdoors, were almost immediate across Africa following the announcement of the first case of Covid-19 case in Egypt on February 14, 2020. Egypt was the first African country to register a covid-19 infection following the pronouncement of Covid-19 as a pandemic^36^. Considering that this was an imported case, several African countries quickly took drastic measures to prevent the cases from getting into their territories, and for countries in which the virus had already entered to prevent further community spread.

There were some countries such as Nigeria and Ghana that did not act fast; they both recorded their first imported cases on February 28^th^ and March 12^th^, 2020, respectively. Countries such as Lesotho declared a state-of-emergency and closed schools on 18th March 2020 following it up with a three-week lockdown even before the first case of Covid-19 was recorded in the country on May 13, 2020. Clearly, some African governments were learning from the success stories of countries like New Zealand, which had taken similar drastic measures immediately, hence preventing the importation of the virus into their territories.

These efforts from governments in taking such timely and sometimes harsh measures collectively contributed to the overall lower rate of spread of the Covid-19 in Africa. This is contrary to European and in the Americas, specifically the UK, Italy, France, and USA, were the economy seemed to have been prioritised over health. The very high mortality rates in these countries, compared with that of any country in Africa, despite their technological advancements affirm the importance and efficacy of the above stringent, sometimes drastic approaches in preventing the spread of Covid-19.

## Following the WHO's recommended guidelines

Several countries that recorded cases of Covid-19 further instituted strict public health and social measures, in line with the WHO recommended protocols, to curtail the spread of the virus. Unlike in some European countries and the United States which saw some public disapproval and demonstrations against observing some of the protocols, especially the wearing of face masks, African citizens embraced the directives from their centralized governments as health and safety necessities and complied with the directives without seeing the restrictions as impeding on their human rights.

The limitation of advanced healthcare and health facilities in most African countries, that could have led to the continent being overwhelmed with Covid-19 infections, somehow appeared to be a blessing in disguise. Citizens accepted and sought out preventive measures, rather than a cure within such restrictive health care systems. Africans are aware of the limitations of their health care systems, in relation to the necessary resources to manage day-to day health needs, albeit any severe cases of Covid-19 requiring ICU treatment. Further, as African governments intensified efforts to implement restrictions curtailing the spread of Covid-19, citizens, made efforts not to contract the virus even though there were still reported cases with their own limited resources and knowledge. In Ghana for example, several people who could not afford imported face masks improvised by using personal handkerchiefs and other clothing materials as face masks. Local seamstresses in most African countries repurposed their businesses by sewing affordable, locally designed cloth-based reusable masks, instead of waiting on central government to distribute “free” face masks or imported ones.

## Innate immunity and the dirt theory

As noted by Gupta and Dutta (2017) people with improved sanitation and diet have less diverse microbiota than their rural, more primitive counterparts 37. There is minimal use of antibacterial and antiviral soaps across most African countries and most African countries cannot boast of great sanitation conditions. As a result of this poor sanitation, the microbiota of most Africans are high and diverse; and this may have provided a quasi-immunity against the coronavirus.

Africa is also home to a lot of herbal medicines and medications for all kinds of illnesses and conditions, including boosting of one's immunity. The use of herbal medicines and medications as well as increasing the intake of specific vegetables, fruits (such as lemons), and spices (like garlic and onions.) as prophylaxis for Covid-19 became very common. Although several of these herbal preparations may have no scientific bases for their usage, most Africans have strong belief in the efficacy of these home-made and herbal medications, thereby intensifying their usage in the advent of Covid-19. It was therefore not surprising when Madagascar, in its early incidence of Covid-19 pandemic outbreak, announced to the world of herbal preparation purported as a cure, despite not being ‘scientifically’ proven. However, with evidence of some food supplements and herbal medicines for the suppression of host antiviral and inate immune response^38^, it may be argued that Africa's attempt to rely on some of its indigenous medical knowledge may have also helped in boosting immune systems, to at least curb the spread of the virus. This further curbed the reliance of Africans on western medicine, rather than indigenous African medicines as observed since colonization. Hence a shift in modern African health care seeking practices, further influenced by modern communication technologies.

## Lessons learned from the Ebola epidemic

Previous experience from effectively managing the Ebola epidemic, which was even more deadly than Covid-19, had provided African countries with experience to manage and curtail the spread of Covid-19 though SARS-COV-2 may have hit African countries by surprise. Notable among these were the efforts by researchers at the Centre Pasteur Institute in Senegal, which resulted in the invention of ‘real time test kits’ for Covid-19 costing less than 1 USD, allowing everyone who wanted to get tested, to be tested. Similarly, ventilators that cost less than 60 USD were quickly developed to cater for severe cases of Covid-19 on the African continent, rather than relying on imported ventilators that could cost tens of thousands of dollars each. These efforts clearly suggest that, contrary to popular believe, Africans understand their health care system gaps; Africa has the technical know-how; Africa has the capacity to conduct high-impact scientific research including health equipment manufacturing. In fact, there are ongoing efforts between the African Researchers Universities Alliance, led by Makerere University in Kenya, the University of Ghana, and Witwatersrand University, to utilize their collective scientific capacity to develop vaccines, including for Covid-19.

## Compliance by the citizens and public measures

The role of citizens in the management and prevention of the spread of the Covid-19 virus cannot be underestimated. While sections 3.1 to 3.6 above, were essential in curbing the spread of Covid-19 across Africa, the compliance of citizens and their efforts at the lowest levels, to ensure safe self-distancing measures must be emphasised. For example, individual self-distancing measures at public gatherings, markets and other unsupervised community settings combined, assisted Africa to emerge as a continent with strengths to build on.

Citizens' contribution through the spread of health promotion messages to members of their families, friends, and communities, formally or informally, cannot be ruled out. The use of modern communication technologies and their applications enhanced the education process, despite other handicaps such as availability of mobile phones and computers. While this is beyond the scope of the current paper, their role, both positive and negative, cannot be overlooked.

## Recommendations

Africa successfully curbed the devastation envisaged by many to plague the continent following the Covid-19 pandemic outbreak during the first wave of the pandemic. However, there is an urgent need for Africa to rethink the tools for managing this and future pandemics beyond rhetoric and continuous hope for handouts from the developed world. Harnessing Africa's human and mineral resources to furnish its health care systems, is necessary and warranted.

Valuing and using Africa's traditional knowledge and medicines should be researched and promoted particularly in light of the covid-19 vaccine inequities. Innovation should be encouraged and supported. Africa's perception that whatever comes from the West, is better will not only ensure that the continent continues to be reactive instead of being proactive, but to even hope for the West to solve Africa's problems will fail Africans today and future generations.

Africa needs to solve its own problems, and this will include leading its own efforts to vaccine development in the future. This will further require African governments to commit to growing science leadership in Africa; developing key research policies and fostering high impact research works in Africa by Africans and providing adequate resources and infrastructure for African researchers. Collaborations between scientists should be encouraged and supported financially by the African governments and institutions.

It is possible that Africa has not been in the race for Covid-19 vaccine development because the continent is already plagued with several killer diseases to which current attention has largely been focused. It may therefore be necessary that African countries embrace and develop their own Covid-19 vaccines on the continent, if they are to eradicate the virus, while instituting appropriate structures to make it possible for Africa to develop its own vaccines in the future.

Despite the ongoing global efforts in fighting the Covid-19 pandemic, it has become clearer that although the disease may remain with us forever or the next few more years, the hope to ensure lasting, life-saving therapies relies on an effective vaccine. As already discussed in the preceding sections, Africa has a lot of science capacity, especially in dealing with viral diseases, epidemiology, use of vaccines, etc. It is this capacity that has helped Africa deal with the Covid-19 pandemic better than other parts of the world. Nevertheless, Africans are counting on the western world to furnish it with these life-saving vaccines.

More research on the second wave of Covid19, and vaccine development in Africa, is required.

## Conclusion

The pandemic nature of Covid-19 and it is management, highlighted the success of Africa drawing on public health measures, despite limited modern health care systems. Covid-19 has sparked widespread research on the theories about the origin, the mode of transmission, and treatment of the novel corona virus or Covid-19. Most researchers posit that the only hope is the development of efficacious vaccines. The announcement and roll out of the two authorized COVID-19 vaccines (from Pfizer Inc. with partner BioNTech SE, Astra Zeneca and Moderna Inc.) in the United Kingdom, Australia and the United States of America amidst vaccine distribution and adminsitration challenges, have provided some semblance of hope to to the world of the proverbial light at the end of the tunnel, this has accentuated the divide in vacine access between rich and poor nations.

The relative lower infection rate and active cases of Covid-19 across the African continent with subpar health care and health systems compared to Europe and North America present a strong case in assessing and rethinking the tools to manage Covid-19 and and future pandemics. Critical research that focuses on the impact of climate factors, youthful population, innate immunity, and strict centralized-implementation of Covid-19 protocols and the rate of COVID-19 cases in Africa could provide useful and timely information in managing Covid-19 and future pandemics.

Preventative approaches that address physical social distance, hand washing, sanitization of surfaces, wearing of face masks, testing, contact tracing, isolation of affected persons; and vaccination remain the tools of choice in managing Covid-19 pandemic worldwide. African leaders, must therefore, avoid complacency, demonstrate proactiveness and preparedness to build the necessary capacity of regional and local public health authorities to design and implement disease-control measures and create supply chain systems in the distribution and administering of Covid-19 vaccines to African population.
